# Variability of high risk HPV genotypes among HIV infected women in Mwanza, Tanzania- the need for evaluation of current vaccine effectiveness in developing countries

**DOI:** 10.1186/s13027-016-0097-2

**Published:** 2016-08-19

**Authors:** Fridolin Mujuni, Mariam M. Mirambo, Peter Rambau, Korn Klaus, Muller Andreas, Dismas Matovelo, Mtebe Majigo, Christa Kasang, Stephen E. Mshana

**Affiliations:** 1 Department of Obstetrics and Gynecology, Weill Bugando School of Medicine, P.O.Box 1464, Mwanza, Tanzania; 2 Department of Microbiology and Immunology, Weill Bugando School of Medicine, P.O.Box 1464, Mwanza, Tanzania; 3 Department of Pathology, Weill Bugando School of Medicine, P.O.Box 1464, Mwanza, Tanzania; 4 Institute of Clinical and Molecular Virology, Enlargen University, Schlossgarten 4, 91054 Erlangen, Germany; 5 Medical Mission Institute, Salvatorstrasse 7, 97067 Wuerzburg, Germany; 6 Department of Microbiology and Immunology, Muhimbili University of Health and Allied Sciences, P.O. Box 65001, Dar es Salaam, Tanzania

**Keywords:** HPV, HIV, High risk genotypes, Mwanza

## Abstract

**Background:**

High risk (HR) human papilloma Virus (HPV) genotypes have been associated with cervical cancer. In Tanzania there is a limited data on the epidemiology of HPV and genotypes distribution among HIV infected women. Here we document varieties of HPV genotypes associated with cervical squamous intraepithelial lesions (SIL) among HIV- infected women at Bugando Medical Centre, Mwanza-Tanzania.

**Methods:**

A cross sectional hospital based study involving HIV infected women was conducted between August and October, 2014. Exfoliated cells from ectocervix and endocervix were collected using cytobrush. HPV genotypes were detected using polymerase chain reaction (PCR) followed by sequencing using specific primers targeting broad range of HPV types. Cytology was done to establish squamous intraepithelial lesions. Log binomial regression analysis was done to establish risk ratios (RR) associated with HPV infection using STATA version 11.

**Results:**

A total of 255 HIV infected women with mean age 39.2 ± 9.1 years were enrolled in the study. HPV DNA was detected in 138/255 (54.1 %, 95 % CI: 47-60) of HIV infected women. Twenty six genotypes were detected in various combinations; of these 17(65.3 %) were of HR genotypes. HR genotypes were detected in 124(48.6 %) of HIV infected women. Common HR genotypes detected were HPV-52(26), HPV-58(21), HPV-35(20) and HPV-16(14). The risk of being HPV positive was significantly higher among women with CD4 counts <100 (RR: 1.20, 95 % CI: 1.05-1.35, *P* = 0.006) and women with SIL (RR: 1.37, 95 % CI: 1.11-1.68, *P* = 0.005)

**Conclusion:**

Significant proportion of HIV infected women with low CD4 counts have various grades of cervical SIL associated with varieties of uncommon HR genotypes. There is a need to evaluate the effectiveness of the current vaccine in preventing cervical cancer in developing countries where HIV is endemic.

## Background

Human papillomavirus (HPV) is a very common sexually transmitted infection which is acquired through body fluids and skin-to-skin contact [1]. It has been known as the causal agent of cervical cancer [2]. In sub-Saharan Africa, the incidence of cervical cancer has reached up to 40.7 cases per 100,000 women and it is the second cause of death among cancers affecting women [3, 4]. In HIV infected women the risk of acquiring HPV is even higher due to immune suppression caused by HIV infection which has the same epidemiological pattern as HPV in sub-Saharan Africa [5, 6]. In most of these cases infection with multiple genotypes has been found to predominate [5, 7–13].

Using molecular techniques, different HPV genotypes have been identified and categorized as high risk (HR) genotypes that are considered to be carcinogenic. These include HPV- 16, 18, 31, 33, 35, 95, 45, 51, 52, 56, 58, 59, 68, 73 and 82; and are considered to cause approximately 95 % of cervical cancers [14]. Human papilloma virus genotypes 6, 11, 40, 42, 43, 44, 54, 61, 70, 72, 81, and cp 6108 have been classified as low risk (LR); majority of these have been found to cause genital warts [15]. Vaccines for HPV are available in some countries and have been found to be effective in reducing infection and its associated consequences [16].

As reported earlier in Africa, women particularly those who are HIV infected are under-represented in the global estimate of HPV genotypes [17]. This necessitates the need to emphasize HPV screening programs for understanding its epidemiology and effective control. Here we document varieties of HPV genotypes associated with cervical squamous intraepithelial lesions (SIL) and associated factors among HIV- infected women at Bugando Medical Centre, Mwanza-Tanzania underscoring the importance of focused control strategies to prevent cervical cancer in developing countries.

## Methods

### Study design, site and population

A cross sectional hospital based study involving 255 HIV infected women was conducted between August and October, 2014 at Bugando Medical Centre (BMC) HIV care and treatment clinic (CTC). The clinic attends patients who are on antiretroviral therapy (ART) and those who have not started ART. The CTC provides routine Papanicolaou (PAP) smear and gynaecological services with no routine screening of HPV. An average of 80 revisiting HIV infected women are seen per day [18].

### Patient selection, socio-demographic and clinical information

The study included all HIV infected women aged >18 years attending the CTC during the study period while excluding women who underwent wedge resection of the cervix and those with missed CD4 counts within 3 months of specimen collection. Socio-demographic and relevant clinical information collected were number of sexual partners, parity, marital status, age at 1st sexual debut, history of STI, history of genital warts, contraceptive use, and CD4 counts.

### Sample collection and Laboratory procedures

Cervical exfoliated cells from the ectocervix and endocervix were obtained using cytobrush; there after the tip of the cytobrush was placed into a transport medium and stored at -10 °C until processing. Papanicolaou (PAP) smear was also taken, processed and interpreted based on the revised 2001 Bethesda system [19].

### Detection of HPV DNA

After vigorous vortexing of transport media, cytobrush was removed and 200 μl of sample was used for nucleic acid extraction with the MagNaPure LC DNA Large Volume Kit on a MagNaPure LC instrument (Roche Diagnostics, Mannheim, Germany) with elution volume set at 100 μl. For the detection of HPV DNA, two different end-point PCR assays (GEN-1 and GEN-2) were done as previously described [20]. Both assays were done using primers targeting sequences which are conserved among a broad range of HPV genotypes, including all major genital HPV genotypes (Tables 1 and 2). These primers amplify fragments of approximately 460 bp for GEN-1 and 220 bp for GEN-2. All PCR products were sequenced (Eurofins Genomics, Ebersberg, Germany); for sequencing of the GEN-1 PCR products, only primer CP4 was used, whereas for GEN-2 PCR products both PPF1 and PPR2 primers were used as previously described [20]. After editing the sequence files using Vector NTI Advance 9 software (Life Technologies, Darmstadt, Germany), sequences were subjected to BLAST search (http://blast.ncbi.nlm.nih.gov/Blast.cgi) to identify the HPV genotypes. The efficiency of nucleic acid extraction was controlled by a real-time PCR assay from our routine diagnostics simultaneously targeting human cytomegalovirus (HCMV) and the human albumin gene.

**Table 1:** Sequences of primers and probes for HPV detection and controls

Name	Sequence	Target
HPV GEN1 PCR		
CP4	5´-ATG GTA CAR TGG GCA TWT GA-3´	HPV E1 (nt 1942-1961)
CP5	5´-GAG GYT GCA ACC AAA AMT GRC-3´	HPV E1 (nt 2400-2378)
HPV GEN2 PCR		
PPF1	5´-AAC AAT GTG TAG ACA TTA TAA ACG AGC-3´	HPVE1 (nt 2082-2108)
PPR2	5´-ATT AAA CTC ATT CCA AAA TAT GA-3´	HPVE1 (nt 2336-2314)

Numbering is according to the sequence of HPV16W12E (GenbankIDNr. AF125673)

**Table 2:** Socio-demographic & Reproductive characteristics of the study population

Characteristics	Frequency/mean/median	Percentage (%)
^a^Age(years)	39.2 ± 9.1	
^b^ARV duration(years)	3(IQR:1-6)	
Residence		
Rural	158	62.0
Urban	97	38.0
SES		
Low	117	45.9
High	138	54.1
H/STI		
NO	213	83.5
YES	42	16.5
Parity		
Nullporous	24	9.4
≥ 1 child	231	90.6
Level of education		
Lower level	209	81.7
High level	46	18.3
CD4 cells/μl		
> 200	134	52.6
200-100	99	38.8
< 100	22	8.6
Age at first intercourse		
Below 18	181	71.0
Above 18	74	29.0
Contraceptive use		
None	164	64.3
Hormonal	62	24.3
Condom	29	11.4
Marital status		
Not married	144	56.5
Married	111	43.5
ARV use		
None	24	9.4
≤ 6 months	23	9.0
> 6 months	208	81.6

^a^mean, ^b^median

### Data analysis

The data were entered in the computer using Microsoft excel software and analysed using STATA version 11(STATA Corp LP, USA). Continuous variables were summarized as mean with standard deviation or as median with interquartile range whilst categorical variables were summarized as proportions. Socioeconomic status (SES) was defined using education, employment and business status of the participant. Log binomial regression for estimation of relative risk ratios was done followed by log multinomial regression analysis for the factors with *P* value of <0.2. Cross tabulation was done to detect variables with collinearity. Factors found with collinearity were not subjected into log multinomial regression analysis. The chi square test and fisher’s exact test were done to compare the distribution of HPV genotypes and SIL. Women with mixed genotypes were regarded as having HR genotype if HR genotype was present. Level of significance was measured by *p*-value whereby factors with *p*-values of less than 0.05 were considered statistically significant.

## Results

### Sociodemographic and obstetric characteristics

The mean age of enrolled women was 39.2 ± 9.14 years. A total of 111(43.5 %) were married and 46 (18.03 %) and 209(81.7 %) had higher and lower education respectively. Majority of the enrolled women 138(54.1 %) had high socioeconomic status. A total of 158(61.9 %) were residing in rural areas (Table 3).

**Table 3:** Genotypes distribution of HPV

HPV genotypes	Frequency	LR/HR
HPV 6	1	LR
HPV 16	14	HR
HPV 18	8	HR
HPV 31	9	HR
HPV 33	4	HR
HPV 34	1	HR
HPV 35	20	HR
HPV 40	2	LR
HPV 42	5	LR
HPV 45	10	HR
HPV 47	1	LR
HPV 51	3	HR
HPV 52	26	HR
HPV 53	5	HR
HPV 54	2	LR
HPV 56	5	LR
HPV 58	21	HR
HPV 59	3	HR
HPV 66	5	HR
HPV 68	2	HR
HPV 73	1	HR
HPV 74	1	LR
HPV 82	4	HR
HPV 83	3	LR
HPV 90	3	LR
HPV 103	1	Unclassified
Total	160	

### Prevalence of HPV

Of the 255 enrolled women, 138 (54.1 %, 95 % CI: 47-60) were found to have HPV DNA in their cervical cells. Out of 138 HPV infected women; 124 (48.6 %) were found to be infected with HR genotypes.

### Genotypes distribution

In 138 HPV infected women; 26 genotypes were detected in various combinations. A total of 17/138(12.3 %) of HIV infected women had multiple HR genotypes. Of 26 genotypes, 17(65.4 %; 95%CI: 44.7-81.2) were HR while 9 (34.6 %, 95%CI: 14-49) were LR genotypes (*p* = 0.028). High risk genotypes detected were 16, 18, 31, 33, 34, 35, 45, 51, 52, 53, 56, 58, 59, 66, 68, 73 and 82 whereas LR genotypes detected were 6, 40, 42, 47, 54, 74, 83 and 90. The total frequency of the genotypes detected was 160 (Table 4); of these common HR genotypes were HPV-52(26, 16.3 %), HPV-58 (21, 13.1 %), HPV-35(20, 12.5 %) and HPV-16(14, 8.8 %).

### Cervical squamous intraepithelial lesions (SIL) and genotypes distribution

**Fig. 1:**
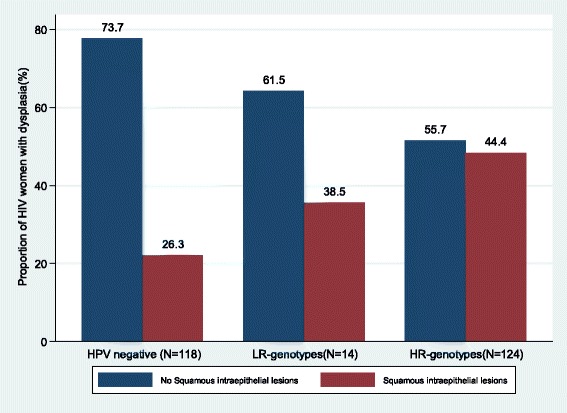
High risk and low risk HPV genotypes in relation to cervical squamous intraepithelial lesions

Out of the 255 women; 91(35.6 %) had SIL on cytological studies. Of 91 HIV infected women with SIL, 74 (81.3 %) had low grade SIL whilst 17(18.7 %) had high grade SIL. Eleven (64.7 %) of women with high grade SIL had positive HPV DNA in their cervical cells. Amongst these eleven women none-16 and non-18 genotypes were detected in 9(81.8 %) of them. Out of 124 women infected with HR genotypes, 55 (44.4 %) had any kind of SIL compared to only 31 (26.3 %) of 118 women with no HPV infection (*P* = 0.003) Fig. 1. Non-significant association between LR genotypes and SIL (5/14 vs. 31/118, *P* = 0.264) was observed. Of 17 women with multiple high risk genotypes, 1(5.8 %) had high grade SIL, 9(52.9 %) had low grade SIL and 7(41.2 %) had no SIL.

### Factors associated with HPV positivity among HIV infected women

On log binomial regression analysis; women with good socioeconomic status, low CD4 counts, use of ARV ≤6 months and presence of SIL significant high risk of HPV infection as shown in Table 4. Only women with low CD4 counts (RR: 1.2, 95 % CI: 1.05-1.35, *P* = 0.006) and presence of SIL (RR: 1.37, 95 % CI: 1.11-1.68, *P* = 0.005) remained with significant higher risk of being infected with HPV on log multinomial regression analysis.

**Table 4:** Log binomial and log multinomial regression analysis on the factors associated with HPV positivity among HIV infected women

Characteristics	HPV positivity %	Log binomial RR(95 % CI)	*p* value	Log multinomial RR(95 % CI)	*p*-value
Age					
> 45 (59)	25(42.4 %)	1			
≤ 45 (196)	113(57.6 %)	1.3 (.98- 1.87)	0.06	1.26(0.89-1.4)	0.266
Residence					
Rural(158)	85(53.8 %)	1			
Urban(97)	53(54.64 %)	1.01(0.80-1.28)	0.896		
SES					
Low(117)	54(46.2 %)	1			
High (138)	84(60.9 %)	1.31(1.04-1.67)	0.022	1.24(0.97-1.56)	0.064
H/STI					
NO (213)	111(52.11 %)	1			
YES (42)	27(64.29 %)	1.23(0.95-1.599)	0.113	1.15(098-1.6)	0.061
Parity					
Nullporous (24)	14 (58.33 %)	1			
≥ 1 child (231)	124 (53.68 %)	0.92(0.64-1.32)	0.650		
CD4					
> 200 (134)	67(50.00 %)	1			
200-100(99)	54(54.55 %)	1.09(0.85-1.39)	0.490		
< 100 (22)	17(77.27 %)	1.54(1.16-2.05)	0.003	1.20(1.05-1.35)	0.006
Age at first intercourse					
Below 18 (181)	96(53.04 %)	1			
Above 18 (74)	42(56.76 %)	1.07(0.84-1.36)	0.582		
Marital status					
Not married (144)	76(52.78 %)	1			
Married (111)	62(55.86 %)	1.05(0.84-1.32)	0.623		
^a^ARV use					
> 6 months(208)	117(51.44 %)	1			
≤ 6 months(23)	16(69.5 %)	1.28 (1.00-1.036)	0.049		
SIL					
Absent (164)	78(47.5 %)	1			
Present (91)	60(65.9 %)	1.49(1.18-1.87)	0.001	1.37(1.11-1.68)	0.005
Contraceptive use					
None (164)	87(53.5 %)	1			
Hormonal (62)	32 (51.6 %)	0.9729(0.73-1.288)	0.848		
Condom(29)	19 (65.5 %)	1.23(0.914-1.66)	0.219		

^a^ARV use was not subjected into multivariate analysis because of its collinearity with CD4 counts,

## Discussion

Infection with oncogenic or HR human papillomavirus (HPV) genotypes is the major cause of cervical cancer. With the introduction of the quadrivalent HPV vaccine which prevents four HPV types: HPV 16 and 18, as well as HPV 6 and 11, that cause 90 % of genital warts the knowledge concerning the distribution of HPV genotypes has become increasingly public health concern [21]. This study for the first time in Mwanza, Tanzania; documents high proportion of HIV infected women carrying varieties of HR genotypes similarly to previous reports [6, 22, 23]. As in previous studies [24], a significant proportion of women were uncommonly infected with multiple HR genotypes. These findings are in agreement with previous studies which reported uncommon HR genotypes infecting HIV infected women in developing countries [4, 22, 25, 26]. This calls for the need to include uncommon high risk genotypes in the current vaccine production especially for areas where HIV is endemic. The recently introduced a 9 valent (nanovalent) vaccine which includes HPV 52, 58, 45[27]; the commonest genotypes detected in this study is of great advancement especially in sub Saharan Africa where HIV is endemic. The World health organization (WHO) should consider advocating replacing the current quadrivalent vaccine which is available in many centers in developing countries with nanovalent vaccine. One of the interesting findings in this study is the presence of HPV 35 genotype in relative high frequency which has been also found in other African countries [22, 26, 28]. Unexpectedly, the recent introduced nanovalent vaccine does not include HPV 35 genotype emphasing the need to use epidemiological data for future vaccine development.

Variation of HPV genotypes can occur even within the country which might have implication in vaccine efficacy in the same country. As evidenced by the fact that in the current study, HPV-16 was not the commonest genotype among women with high grade SIL while in the previous study in Tanzania among HIV and non HIV infected women HPV-16 was the commonest genotype among women with high grade SIL [12]. There is a possibility that the current existing HPV vaccines might have diverse efficacy especially in developing countries.

The current study confirms the clear association between HPV infection and SIL. As in previous studies [29–31], it was observed that women infected with HR genotypes were more likely to have SIL than non-infected and those infected with LR genotypes. Though none of the women with LR genotypes had high grade SIL; it was observed that 35.7 % of women infected with LR genotypes had low grade SIL, following these women over time to determine cervical changes and acquisition of other genotypes will provide important information regarding the role of LR genotypes and SIL. In addition, women with low CD4 counts (<100) had significantly higher risk of acquiring HPV infection (low and high risk genotypes) as compared to their counterparts. Our findings are similar to what have been reported earlier [11, 32–34]. This is due to immune suppression which poses the risk of opportunistic infections including HPV.

The major limitation of this study is inability to perform HIV viral load which could be used to correlate virological failure and HPV infection. In addition some important information such as tobacco use and information for those who declined were not collected. However the study established HPV prevalence and genotypes distribution among HIV infected women.

## Conclusion

In present study, HIV seropositive women with low CD4 counts and various grades of cervical SIL are significantly infected with HR HPV genotypes. In countries where HIV is endemic the governments should consider addition of HPV vaccine in national immunization programme focusing on epidemiology of HPV genotypes. Data on the efficacy of the current vaccine in preventing SIL/cancer and warts are highly needed in developing countries for appropriate policy decision making.

## Abbreviations

ART: Anti-Retroviral Therapy
BMC: Bugando Medical Centre
CD4: Cluster of Differentiation type 4
CTC: Care and Treatment Clinics
CUHAS: Catholic University of Health and Allied Sciences
HAART: Highly Active Ant-Retroviral Therapy
SIL: Squamous Intraepithelial Lesions
HIV: Human Immunodeficiency Virus
HPV: Human Papilloma Virus
HR: High Risk
LR: Low Risk
RR: Risk ratio
SIL: Squamous Intraepithelial Lesion
SES: Social Economic Status
WHO: World Health Organization
